# Chondroitin Sulfate Proteoglycans: Structure-Function Relationship with Implication in Neural Development and Brain Disorders

**DOI:** 10.1155/2014/642798

**Published:** 2014-05-14

**Authors:** Speranta Avram, Sergey Shaposhnikov, Catalin Buiu, Maria Mernea

**Affiliations:** ^1^Anatomy, Animal Physiology and Biophysics Department, Faculty of Biology, University of Bucharest, 91-95th Independentei Street, 050095 Bucharest, Romania; ^2^Norgenotech AS, Totenvegen 2049, 2848 Skreia, Norway; ^3^Automatic Control and Systems Engineering Department, Faculty of Automatic Control and Computers, “Politehnica” University of Bucharest, 313th Independentei Street, 060042 Bucharest, Romania

## Abstract

Chondroitin sulfate proteoglycans (CSPGs) are extracellular matrix components that contain two structural parts with distinct functions: a protein core and glycosaminoglycan (GAG) side chains. CSPGs are known to be involved in important cell processes like cell adhesion and growth, receptor binding, or cell migration. It is recognized that the presence of CSPGs is critical in neuronal growth mechanisms including axon guidance following injury of nervous system components such as spinal cord and brain. CSPGs are upregulated in the central nervous system after injury and participate in the inhibition of axon regeneration mainly through their GAG side chains. Recently, it was shown that some CSPGs members like aggrecan, versican, and neurocan were strongly involved in brain disorders like bipolar disorder (BD), schizophrenia, and ADHD. In this paper, we present the chemical structure-biological functions relationship of CSPGs, both in health state and in genetic disorders, addressing methods represented by genome-wide and crystallographic data as well as molecular modeling and quantitative structure-activity relationship.

## 1. CSPGs Structure-Biological Activity Relationship in Health State

Over the past decade many studies were focused on the complex interactions between neuron and glia [1–4] and the general opinions of these studies were that extracellular matrix molecules (ECM) like CSPGs are involved in the synapsis process among neurons and astrocytes.

The neural ECM include the CSPGs of the lectican family [1, 4] as well as glycoproteins such as the tenascins [5]. Lecticans, a family of hyaluronan binding proteoglycans, are represented by aggrecan (ACAN), versican (VCAN), neurocan (NCAN), and brevican (BCAN) [4, 6] and have an important role in neuronal growth mechanisms represented by neuroplasticity, axon guidance, or neuron repair processes following injury to the spinal cord or brain [4, 7–9]. It was shown that CSPGs levels fluctuate during the brain development process, as high values were detected in immature brain of embryos but not in the mature brain of normal adults [6, 10, 11]. During postnatal neural development, CSPGs play an active role in axon guidance, with the movement of axons being established through second messengers such as calcium and cyclic nucleotides [7].

Structurally, CSPGs contain a protein core covalently linked to one or more unbranched GAG chains that play different roles in CSPGs biological functions [12, 13]. Several studies have emphasized the important roles of GAG chains in the growth-inhibitory and neuron regenerative processes [1, 12, 14–16]. Also, it was indicated that most functions of CSPGs are predominantly performed by the chondroitin sulfate moieties, while the core proteins act as a scaffold [12]. Even if it was supposed that* in vivo* the CSPG core proteins do not play a critical role, several very recent studies showed that CSPG core proteins inhibit neurite outgrowth and changes in expression of these proteins genes lead to psychiatric disorders [17–19]. Iseki et al. [20] studied the functional roles of CSGPs protein core and they noticed the following: (i) CSGP type NG2 is a major inhibitor of axonal growth* in vitro*; (ii) VCAN isoform V2 inhibits neurite outgrowth in the central and peripheral neurons, while (iii) BCAN is an inhibitor of neurite outgrowth and (iv) BCAN and NCAN mRNAs are upregulated in astrocytes in central nervous system glial scars.

Biosynthesis and degradation process of CSPGs were studied in detail by Kitagawa et al. [21, 22], Laabs et al. [12], Izumikawa et al. [23, 24], and recently Mikami and Kitagawa [25]. In these studies it was shown that the biosynthesis of CSPGs is a very complex process that requires the presence of the entire enzymatic machinery, broadly distributed in tissues, including the brain [12, 21–26]. Enzymes are represented by glycosyltransferases, glucuronyltransferase-I, chondroitin synthase family of enzymes, and chondroitin polymerizing factor [12, 21–26]. Some of chondroitin sulfate enzymes and their chromosomal location and abbreviation are presented in Table 1 [25]. The entire mechanism of CSGPs action in central nervous system is unknown, but several studies [20, 27] showed that, at axonal level, the removal of GAG chains by chondroitinase ABC allowed some axonal regrowth.

NCAN is a significant component of the ECM, and its level is modulated by many factors. Important details about the structural features of NCAN were presented in previous studies [12, 18, 28–32] which described the complete coding sequence of the human NCAN mRNA as well as mapping data, expression analysis, and genomic structure. Investigation of NCAN gene conservation among several eukaryotic species [33] indicated a close homology between sequences of NCAN from* Homo sapiens *(human),* Pan troglodytes *(chimpanzee),* Macaca mulatta *(Rhesus monkey),* Canis lupus *(dog),* Bos taurus *(cattle)*, Mus musculus *(house mouse)*, Rattus norvegicus *(Norway rat), and* Gallus gallus *(chicken). The amino acid sequence of human NCAN shows 63% identity with both the mouse and the rat sequences [28].

Structural studies on NCAN [12, 18, 28–30] revealed the following: (i) full-length NCAN core protein from human species comprises 1321 amino acids with a molecular weight of 220 kDa; (ii) NCAN gene is found on chromosome 19p12; (iii) NCAN contains 5 to 6 N-linked and up to 40 O-linked oligosaccharides; (iv) NCAN presents three active domains, namely, a N-terminal hyaluronan binding domain, a C-terminal lectin-like domain (CLD), and a central GAG attachment region that has no homology with other family members.

**Figure 1:**
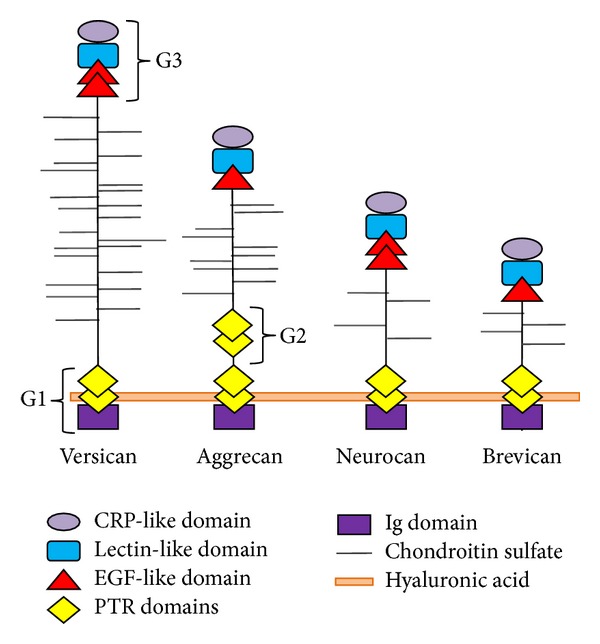
Domain structure of human VCAN, ACAN, NCAN, and BCAN. The schematic representation was performed based on information retrieved from UniProt database [34], entries P13611 (human VCAN sequence), P16112 (human ACAN sequence), O14594 (human NCAN sequence), and Q96GW7 (human BCAN sequence). The four CSPGs present the following: (i) an N-terminal G1 domain comprising an immunoglobulin-like domain (Ig domain) and proteoglycan tandem repeats (PTR) domains involved in binding hyaluronic acid, (ii) a chondroitin sulfate attachment region, (iii) a C-terminal G3 domain comprising epidermal growth factor- (EGF-) like domains, a lectin-like domain, and a complement regulatory protein- (CRP-) like domain. The G2 domain, specific only for ACAN structure, comprises two PTR domains.

It has been shown that members of lectican family are characterized by similarity in N-terminal globular hyaluronan binding domains and CLDs but differ considerably in their central regions [35] which may induce different biological functions for lectican family members. The domain structure of ACAN, VCAN, NCAN, and BCAN is presented in Figure 1.

The hyaluronan binding domain, common in all four lecticans [36], is especially important during inflammatory process and tumor metastasis [37], while the CLDs are currently involved in processes like cell-cell adhesion [38] and immune response to pathogens [39–41].

Studies [30–32, 36, 41–44] focused on the functional features of NCAN showed that it binds to various ECM components, such as hyaluronan, heparin, tenascin-C, and tenascin-R, it is able to modulate the cell binding and neurite outgrowth, and it can also interact with neural cell adhesion molecule NCAM.

The interaction between the CLD of NCAN and tenascin-C active domain was investigated by Rauch et al. [30] where the authors concluded the following: (i) NCAN CLD interacts with the pair of tenascin-C FnIII domains, namely, TNfn4 and TNfn5, in a divalent cation-dependent manner and (ii) NCAN-tenascin-C complex is able to modify the functional properties of tenascin-C.

**Figure 2:**
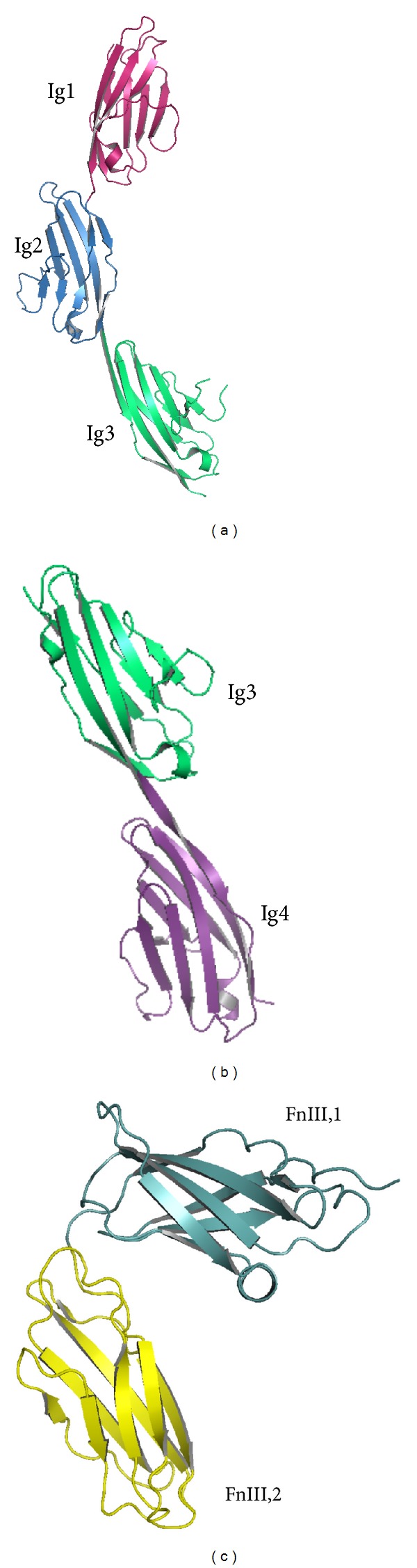
The crystal structures of the following NCAM fragments: (a) the first three N-terminal Ig modules (Ig1, Ig2, and Ig3), according to the crystal structure 1QZ1 [49]; (b) the third and fourth Ig modules (Ig3 and Ig4), according to the crystal structure 2XY1 [50]; and (c) first two fibronectin type III modules (FnIII,1 and FnIII,2), according to the crystal structure 2VKW [51]. The domains are labeled in the figure and are represented using different colors. Figures were made by Mernea M. using the free license software Pymol [52].

Another important partner of NCAN is NCAM [31, 32], the first described member of cell adhesion receptors from the immunoglobulin superfamily. NCAM extracellular domain comprises five immunoglobulin (Ig) modules and three fibronectin type III modules (see Figure 2). Rao et al. [45] and Kasper et al. [46] showed that the Ig modules of NCAM are critical for many features of NCAM like the hemophilic interaction of NCAM, affinity for the chondroitin sulfate binding site [47], and the interaction with oligosaccharides [48]. In order to elucidate the NCAN-NCAM mechanism of interaction, Rauch et al. [31] performed a very interesting study whose results showed that two NCAM residues located in the CLD, namely, methionine 773 and leucine 639, are critical for NCAM/NCAN interaction. These amino acids were able to increase NCAM capacity to interact with NCAN, while threonine in 950 reduced the capacity of NCAM to interact with NCAN. The study revealed the following: (i) NCAM interacts with all major NCAN domains in almost similar manner; (ii) NCAN active domains are not able to inhibit NCAM if they are dissociated from each other.

Regarding the brain expression of NCAN during embryonic and postnatal development, it was shown [29, 53, 54] that, in rats, NCAN was first detected at the tenth embryonic day and that it had a maximum expression level around birth and decreased levels in the mature brain [29]. Zhou et al. [29] also showed that NCAN is expressed in several areas like thalamus, spinal cord, hypothalamus, or the cerebellum. Additional information was brought by Rauch et al. [30] which showed that NCAN interacts with tenascin-C in cerebellum at postnatal day 7.

ACAN, another member of the lectican family of CSPGs, is an important constituent of perineuronal nets [55, 56]. It is also distributed in the entire body, mostly in cartilage and brain, as aggregates formed with hyaluronic acid that binds to ACAN N-terminal domain [30]. It plays a major role in growth and homeostasis [57]. The domain structure of ACAN includes an N-terminal domain, namely, G1, separated from a second globular domain (G2) by a short interglobular domain, an elongated domain carrying keratan sulfate and chondroitin sulfate chains, and a C-terminal globular G3 domain [58, 59] (see Figure 1). Specific for G1 domain is an N-terminal immunoglobulin-like repeat and two proteoglycan tandem repeats domains. These proteoglycan tandem repeats domains are responsible for the interaction of ACAN with hyaluronan [60]. It is also shown that G2 domain, found only in ACAN structure, contains two proteoglycan tandem repeats domains but these have no involvement in the interaction with hyaluronan [60]. The G3 domain comprises four structural motifs: two EGF-like repeats, CLD, and a complement regulatory protein (CRP or sushi) repeat [61]. The functions of EGF and CRP motifs of G3 are unknown, while CLD is involved in binding the carbohydrate [62] and in establishing calcium-dependent high affinity interactions with ECM proteins: tenascin-R, tenascin-C, fibulin-1, fibulin-2, and fibrillin-1 [63]. ACAN genes identified as putative homologues belong to the following species:* H. sapiens*,* M. mulatta*,* C. lupus*,* M. musculus*, and* R. norvegicus *[33].

VCAN is another member of lectican family. Four isoforms of VCAN (V0, V1, V2, and V3) have been identified in various tissues, including the brain [64]. Structurally, all VCAN isoforms include an N-terminal domain (G1), a GAG attachment region, and a C-terminal domain (G3) (see Figure 1). The exception is represented by V3 isoform, which has no GAG attachment region [64] but maintains its ability to bind hyaluronan through the G1 domain and to interact with EGF receptors through the EGF-like subdomains of G3 [65]. Similarly to NCAN, VCAN interacts with other ECM components through its three structural domains. Based on the interactions mediated by G1 and G3 domains, VCAN is able to regulate cell invasion and metastasis [66].

The studies presented in this section clearly suggest that, in the body, CSPGs expressions impose their functions and any mutation which appear at nucleotide sequence of CSPGs may induce changes in their functions. In the next section we will present changes in NCAN structure able to induce brain disorders.

## 2. CSPGs Structure-Function Relationship in Genetic Disorders

The expression of NCAN, VCAN, and ACAN in cells was associated with inhibition of axonal regeneration and neurogenesis after central nervous system injury [8, 9, 16, 20, 67–69]. Also, the presence of NCAN mutants was associated with psychiatric disorders as bipolar disorder, schizophrenia, and ADHD [17–19, 70–74].

Studies [8, 9, 16, 20, 67–69] on the levels of NCAN, BCAN, and VCAN after spinal cord injury identified large upregulation of these CSPGs which leads to inhibition of axon growth. The upregulation is obvious as early as one day after injury and the return to normal expression levels occurs in 4 weeks after injury [8].

Many recent studies were focused on genome-wide significant association (GWSA) between variation in the NCAN gene expression and psychiatric disorders such as BD, schizophrenia, and ADHD [17–19, 70–74]. Recently, Mühleisen et al. [17], Cichon et al. [18], Schultz et al. [70], and Oruc et al. [19] performed GWSA studies between common variation in the NCAN gene (NCAN, rs1064395) and the psychiatric disorders BD and schizophrenia.

Mühleisen et al. [17] focused their research on identifying if BD and schizophrenia partly present common genetic risk factors in a large number of subjects (5061 patients and 9655 controls) genotyped for NCAN rs1064395 single-nucleotide polymorphism (SNP). Their results showed that rs1064395 A-allele, known to be a risk factor for BD, was significantly overrepresented in schizophrenia patients compared to control group [17]. These results, sustained by similar data [18, 19, 70, 75], suggest that genetic variation in NCAN rs1064395 represents a common risk factor for BD and schizophrenia. The patients with schizophrenia showed significantly increased rates of BD in comparison with control subjects. Furthermore, the study of schizophrenia elaborated by Mühleisen et al. was performed on independent samples, different from those used by the Psychiatric GWSA Consortium study [76], and showed that BD and schizophrenia share common etiological factors [17].

Schultz et al. [70] recently described the association between NCAN alleles and cortical thickness and folding in a number of 63 schizophrenia patients and 65 controls enrolled in their study. These subjects were genotyped for NCAN (rs1064395) SNP and were diagnosed using magnetic resonance imaging. The study results presented the following: (i) in patients with schizophrenia, NCAN risk represented by AA and AG carriers was found to be associated with higher folding in right lateral occipital and lesser in left dorsolateral prefrontal cortical areas; (ii) in control subjects, no such associations were detected; (iii) no significant association was determined in the case of cortical thickness of either patients or controls. The study results reconfirmed the involvement of NCAN in the visual processing and top-down cognitive activity, with these processes being severely disturbed in patients with schizophrenia.

Another significant GWSA study between NCAN gene markers and BD was performed by Cichon et al. [18]. In this study, authors investigated 499,494 autosomal and 12,484 X-chromosomal SNPs in a huge number of subjects (682 patients with BD and 1300 control subjects) from six different European countries. Interesting results were obtained when the correlation between BD and overexpression of NCAN genes was considered, showing that NCAN marker (rs1064395) is present in patients with BD. The relevant conclusions and perspective of the study were as follows: (i) NCAN is a potential BD susceptibility factor; (ii) localization of NCAN at the level of central nervous system (cortex, hippocampus), NCAN alleles, and BD susceptibility are positively correlated; and (iii) NCAN-deficient mice should be furthermore studied in order to identify the more subtle changes in the cognitive processes that are severely affected in BD, like learning and memories. This idea is based on the observation [29] that maintenance of late-phase long-term potentiation in the hippocampal CA1 region in null mutants is reduced, which leads to mild deficits in learning and memory.

NCAN polymorphism association with BD was also investigated by Oruc et al. [19]. The aim of this study was to determine the relationship between NCAN polymorphism and genetic risk in a population sample from Bosnia and Herzegovina [19]. An important number of subjects represented by 56 patients and 30 healthy volunteers were genotyped for NCAN marker (rs1064395) using direct sequencing method. All three expected genotypes, GG, AG, and AA, were observed in this study. The obtained results were as follows: (i) allele AA appeared in frequencies of 1 in case group in 1 case and 0 in control group, and (ii) allele AG appeared in frequencies of 11 in case group in 5 cases and 0 in control group, while (iii) allele GG appeared in frequencies of 44 in case group in 21 cases and 0 in control group. Based on these results the authors concluded the following: (i) no genetic association was found between risk allele A for NCAN and schizophrenia; (ii) suggestive overexpression of risk alleles was found, but no statistical significance could be determined with standard statistical methods for genetic association analysis.

Very recently, Schimmelmann et al. [72] identified for the first time the risk alleles in children with ADHD and BD. Taking into account the high comorbidity of BD and ADHD, their phenotypic overlap especially in pediatric populations, the high heritability of both disorders, and the cooccurrence in families, the authors studied the possibility of the two disorders to share common genetic risk factors. The GWSA study was performed in 495 ADHD children and 1300 control subjects. No significant association was found between childhood ADHD and single BD risk alleles, but the polygene analysis for BD risk alleles at loci in NCAN, BCAN, and lectin, mannose binding 2-like gene, indicated a higher probability of a BD risk allele carrier to be associated with ADHD.

GWSA studies reviewed here presented the critical importance of NCAN marker (rs1064395 SNP) in identification of BD genetic risk and its possible role in BD association with other psychiatric disorders as schizophrenia and ADHD. Unfortunately, the mechanism by which NCAN (rs1064395) SNP could be involved in these psychiatric disorders is still unclear. Beside the clinical studies,* in silico* methods such as molecular modeling and computational mutagenesis techniques could establish a relationship between chemical structure and function of NCAN. These should bring supplementary knowledge on the mechanism by which single or multiple mutations in NCAN gene are able to induce BD genetic risk.

Even if at this time there are no* in silico* studies on the role of rs1064395 SNP in BD risk, based on our expertise in computational mutagenesis and quantitative structure-activity relationship (QSAR) applied to peptides and proteins [79, 80], we may suggest that the critical molecular features for the biological activity of NCAN native and its mutants should be represented by van der Waals surface and/or solvent accessible surface areas [81, 82] or count of atoms and bound types (e.g., polar and hydrophobic atoms, rigid and rotatable bonds) [83]. The substitution of a NCAN amino acid in a crucial position due to expression of a SNP may be able to change its molecular features, even if in a discreet manner, but sufficiently to induce changes in NCAN functionality during neuronal development.

Beside the elucidation of the mechanism by which CSPGs are involved in psychiatric disorders, another very important aspect of both clinical and* in silico* studies is represented by identification of mechanism by which the ligands are able to reduce CSPGs activity in the nervous system. Accordingly, clinical [76] and* in silico* studies [79, 80] focused on drugs involved in psychiatric disorders treatment as well as in reducing CSPGs (NCAN, ACAN, and VCAN) activity are presented in the next section. Also in the next section we present a brief description of* in silico* methods applied to CSPGs.

## 3. CSPGs Structure-Function Relationship* In Silico* Approaches

*In silico* research in medicine is thought to have the potential to speed the rate of discovery and reduce the need for expensive lab work and clinical trials. QSAR is used to obtain a strong relationship between the experimental biological activity of chemical structures and their structural features. QSAR is of great importancein the design of* de novo* drugs that present improvedpharmacokinetic and pharmacodynamic features with reduced side effects [79–81].

Recently, many research groups have applied QSAR techniques to develop new, more potent psychiatric drugs. An increasingly attractive strategy in pharmaceutical science is to discover new applications for drugs that are already clinically approved. For this aim the QSAR methods is an appropriate method.

In line with this aim, here we present QSAR studies applied to drugs used in schizophrenia treatment, namely, antipsychotics, with dual activities: inhibitory effect on lectican family [84] and also on membrane receptors [77, 85]. Schizophrenia symptoms are generally classified in two classes: positive (e.g., delusions, hallucinations, and disorganization) and negative symptoms (apathy, attention impairment, affective disorder, and several degrees of social impairment) [86]. Severe symptoms comprise psychosis, apathy and social withdrawal, important disturbance of professional skills, less independent life, or bad interpersonal relationships. Recent reports mention an increased number of patients diagnosed with psychoses with depression spectrum [87].

**Figure 3:**
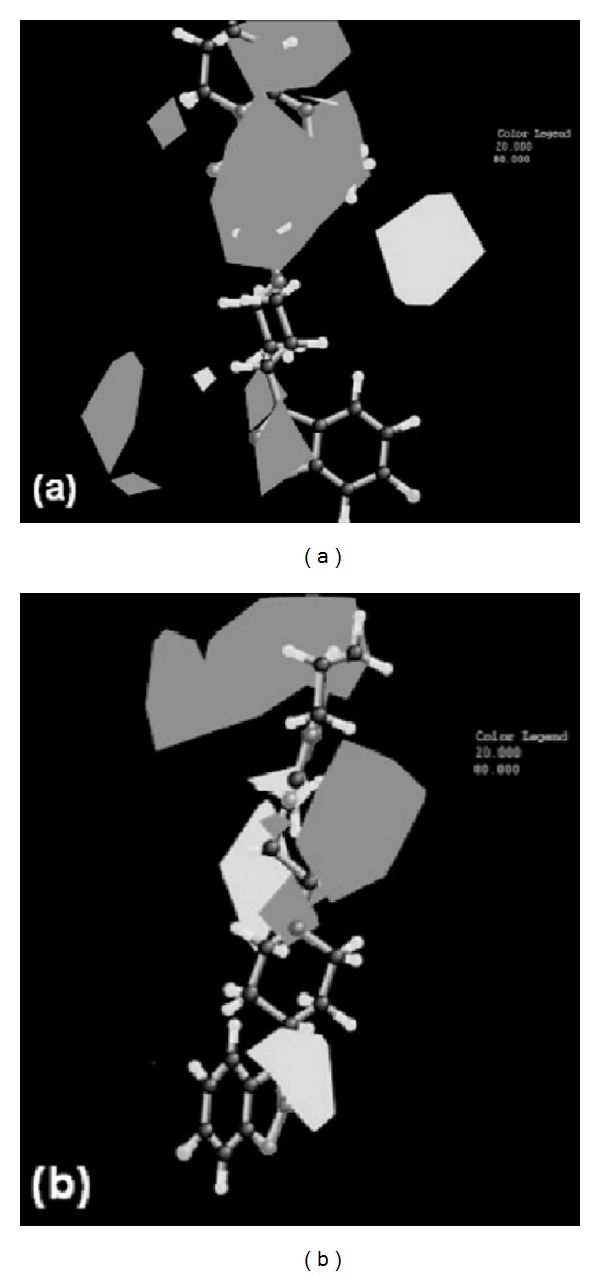
(a) Representation of the favorable (white polygons) and unfavorable (grey polygons) hydrophobic areas of risperidone (biological activity on D2 = 8.18) when its antagonist potency at dopamine D2 receptor is considered and (b) representation of the favorable (white polyhedral) and unfavorable (grey polyhedral) hydrophobic areas of risperidone (biological activity on 5HT2A = 9.76) when its antagonist potency at the serotonin 5HT2A receptor is considered [after Avram et al. [77], copyright permission].

Avram et al. [77, 85, 88] applied advanced 3D QSAR methods to establish the most important descriptors that are critical for antipsychotic drugs interactions with membrane receptors like dopamine D1–D4, serotonin (5HT1A, 5HT2A), or adrenergic. One of these studies [77] has drawn several conclusions: (i) the hydrogen acceptor bond and the hydrophobic, electrostatic, and steric properties should be considered simultaneously to define the antagonist potency at the dopamine D2 receptor; (ii) the hydrogen bond donor and the hydrophobic, electrostatic, and steric properties should be considered simultaneously to define the antipsychotics affinity at the serotonin 5HT2A receptor; (iii) the design of new chemical structures with antagonistic activity on D2 and 5HT2A receptors could be improved by modulating their physicochemical properties, especially their hydrogen bond acceptor/donor and hydrophobic properties (Figure 3). For instance, risperidone derivatives with increased antagonist potency to the dopamine D2 receptor were obtained by increasing hydrophobic contacts on risperidone rings, while simultaneously retaining a fluorine atom [77].

Johnstone et al. [84] studied the antipsychotic drugs from phenothiazines (PhAPs) class such as prochlorperazine, fluphenazine, and trifluoperazine and non-PhAPs that may induce changes in cellular signalling and promote neurite outgrowth on inhibitory substrates. Some of these drugs are able to promote neurite outgrowth in cultured neurons in the presence of inhibitory CSPG substrate. The results revealed very important information: (i) trifluoperazine, prochlorperazine, perphenazine, and fluphenazine significantly enhanced neurite growth, branching, and axon length in hippocampal neurons on the CSPG substrates NCAN, VCAN, and ACAN; (ii) unexpected similar activity of piperazine PhAPs and a novel regeneration-promoting compound named F050 was noticed in increasing central nervous system neuronal outgrowth when growth was restricted by CSPGs; (iii) taking into account the severe side effects induced by PhAP antipsychotics, to use these structures for treatment of central nervous system injury imposes concerning dosage. In this study, for tested PhAP, there was a narrow concentration window between efficacy (growth promotion) and toxicity (cell death). Therefore, the authors suggested that the efforts could include future QSAR studies to identify compounds that promote growth without killing cells.

Other studies [89, 90] were focused on antipsychotics such as olanzapine, quetiapine, and clozapine that were able to enhance growth factor which induced neurite outgrowth in cell line PC12 from adrenal medulla, at concentrations of 10–40 *μ*M [89]. In the same study [89] clozapine and fluphenazine were shown to be able to increase axon lengths from mechanosensory neurons in* Caenorhabditis elegans*, at a concentration of 160 *μ*M [90].

These studies are also relevant to the mechanisms through which antipsychotics reduce the symptoms of psychosis since it was shown that schizophrenia is characterized by deficits in neuronal growth and connectivity [91], and genes associated with schizophrenia, such as disrupted in schizophrenia 1 (DISC1), are known to play significant roles in neurite growth. For example, Pantazopoulos et al. [92] showed that the levels of CSPGs are increased in postmortem schizophrenic brains. Therefore, the ability of antipsychotics to promote growth in the presence of glial-derived inhibitory molecules may represent a mechanism for improving neuronal connectivity in schizophrenic patients.

**Figure 4:**
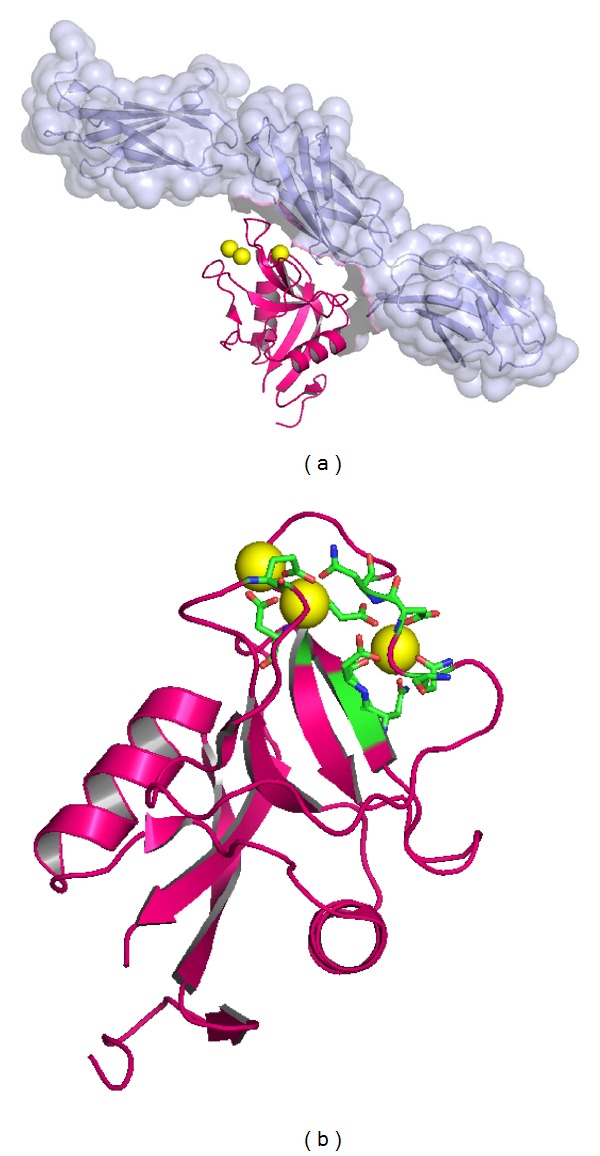
(a) The structure of ACAN core protein in complex with tenascin-R FnIII repeats 3–5, as revealed by the crystal structure 1TDQ [78]. The tenascin-R fragment is represented as a light blue transparent surface that covers its backbone represented with the same color. ACAN core protein is represented with magenta and the three Ca^2+^ ions coordinated by ACAN are represented with yellow. (b) Detail on ACAN core protein (represented with magenta). The Ca^2+^ ions are represented with yellow and the residues involved in coordinating them are represented with green. Figures were made by Mernea M. using the free license software Pymol [52].

Other useful studies for identification of CSPGs structural changes are X-ray crystallography studies supplied by molecular modeling. Such a study was focused on ACAN [56, 78]. The structure of rat ACAN CLD domain was solved by X-ray crystallography (2.6 Å resolution) in a Ca^2+^-dependent complex with fibronectin type III (FnIII) repeats 3–5 of rat tenascin-R [78] (see Figure 4(a)). Tenascin-R and CSPGs present a colocalized expression in the nervous system and form specialized ECM structures called perineuronal nets [93]. Multimeric tenascins act as molecular cross-linkers between CSPG-hyaluronan complexes and the evidence of network formation* in vivo* was obtained by Lundell et al. [78] using electron microscopy. Molecular evidence of this interaction is brought by the crystal structure deposited in Protein Data Bank under the code 1TDQ [78] solved by the same authors. In this structure, ACAN core protein comprises 126 residues and has three disulfide bridges: Cys3-Cys14, Cys31-Cys123, and Cys99-Cys115. The last disulphide bridge is located at 8 Å distance from two Ca^2+^ ions. All residues are numbered according to the fragment in the crystal structure.

The structure 1TDQ comprises a total of three Ca^2+^ ions, with all of them being coordinated by ACAN residues (see Figure 4(b)). The carbohydrate binding Ca^2+^ ion, conserved in all structures of CLDs, is coordinated by amino acids: Gln88, Asp90, Glu97, Asn111, and Asp112 and by a water molecule. A second Ca^2+^ ion is coordinated by amino acids: Glu68, Asn91, Glu97, and Asp98. The third Ca^2+^ ion is coordinated by amino acids Asp98 and Glu68. The last two Ca^2+^ ions are not constant in solved CLDs structures. The Ca^2+^ ions are involved in ordering the neighboring ACAN loops and it was noticed [78] that these loops further make specific interactions with one of the FnIII repeats of tenascin-R.

In complex with the FnIII domain of rat tenascin-R, the *β*6 and *β*7 strands of ACAN and also its extended loop L4 are buried [78]. The contact area between ACAN and tenascin-R is free of carbohydrate. The interaction surface of ACAN is composed of 55% polar and 45% nonpolar residues and can be divided into three regions. Near Ca^2+^ ions, the surface is mostly hydrophobic, with residues Phe92 and Phe93 from L4 loop being of particular interest for the interaction with tenascin. In other CLD structures such as that of mannose binding protein, the homologous residue of Phe92 is an aromatic residue which interacts with the bound carbohydrate [90]. Other residues important for the interaction with tenascin are (i) Val101, Tyr117, and Leu119, which form the hydrophobic pocket that accommodates residue Leu131 from tenascin structure; (ii) residue Asn111 which forms a hydrogen bond with tenascin Leu131; (iii) residue Arg21 which forms two salt bridges with tenascin Glu161; (iv) Asp56 that makes a salt bridge with tenascin Arg183; (v) Asn53 and Asn54 forming hydrogen bonds that block residue Arg260 from tenascin structure; (vi) Trp104 which interacts with a tenascin hydrophobic pocket; and (vii) Gln118 which forms a hydrogen with tenascin Asn181 [78].

## 4. Conclusion and Perspective

We presented here a number of experimental and* in silico* studies focused on characterizing the structural and functional features of CSPGs and their possible inhibitors. All reviewed studies considered the important role of CSPGs (e.g., NCAN, ACAN, VCAN, and BCAN) in physiological cellular processes and also in promoting disorders such as those associated with inhibition of axonal regeneration and neurogenesis after central nervous system injury and psychiatric disorders such as BD, schizophrenia, and ADHD.

Being localized in the neural ECM, these CSPGs have an important role in neuronal growth mechanisms represented by neuroplasticity, axon guidance, or neuron repair processes following injury to the spinal cord or brain. The discussed studies have a major goal to underline the correlation between the structural sequences and/or overexpression of these CSPG and brain disorders. Even if the number of preclinical and clinical studies is significant in this field, the mechanism by which NCAN, VCAN, or ACAN affects human tissues and brain is not enough understood.

For a better explanation of CSPG involvement in cellular processes, recent studies used structural and crystallographic methods as well as QSAR complementary techniques over the experimental methods such as GWSA. These studies were focused on the identification of structural changes underlining the function modification induced by changes in NCAN, VCAN, or ACAN sequences.

In perspective, the simultaneous use of experimental and* in silico* (computational mutagenesis, QSAR on proteins) techniques will allow more reliable early identification of CSPGs genes expression errors and will open the possibility to develop new population screening tests for susceptibility to the development of new and misdiagnosed neurodevelopment disorders.

What is more, by using computational mutagenesis, rational protein design, and the prediction of structures-proteins function relationship, it will be possible to study the structure of NCAN, VCAN, or ACAN mutants which are able or not to induce neurodevelopment disorders.

Considering the already established role of NCAN SNP (rs1064395) as risk marker for BD and schizophrenia disorders, by using QSAR methods we will be able to present the critical molecular features of NCAN SNP (rs1064395) in psychiatric disorders. Besides, QSAR could also be helpful in identifying molecular features of other possible NCAN SNPs markers for psychiatric disorders. On the other hand, these descriptors can be compared with the identical molecular features of native NCAN in order to notice the discreet changes in the structure and functionally of NCAN. We are confident that QSAR is an appropriate method to determine discreet changes in the molecular features of NCAN or other CSPGs.

## Figures and Tables

**Table 1:** Chondroitin sulfate enzymes in human.

Enzymes	Abbreviation	Chromosomal location
Glycosyltransferases involved in synthesis of the tetrasaccharide linkage region		
Xylosyltransferase	XylT	16p12.3
*β*1,4-Galactosyltransferase-I	GalT-I	5q35.2-q35.3
*β*1,3-Galactosyltransferase-II	GalT-II	1p36.33
*β*1,3-Glucuronyltransferase-I	GlcAT-I	11q12.3

Glycosyltransferases involved in synthesis of the repeating disaccharide region of CS chains		
Chondroitin synthase	ChSy-1	15q26.3
	ChSy-2	5q23.3
	ChSy-3	7q36.1
Chondroitin polymerizing factor	ChPF	2q35
Chondroitin transferase	ChGn-1	8p21.3
	ChGn-2	10q11.21

Sulfotransferases and epimerases		
Chondroitin 4-*O*-sulfotransferase	C4ST-1	12q
	C4ST-2	7p22
	C4ST-3	3q21.3
Dermatan 4-*O*-sulfotransferase	D4ST-1	15q15.1
Chondroitin 6-*O*-sulfotransferase	C6ST-1	10q22.1
Uronyl 2-*O*-sulfotransferase	UST	6q25.1
GalNAc 4-sulfate 6-*O*-sulfotransferase	GalNAc4S-6ST	10q26
Glucuronyl C-5 epimerase	DS-epi1	6q22
	DS-epi2	18q22.1

## Abbreviation

ACAN: Aggrecan
ADHD: Attention deficit hyperactivity disorder
BCAN: Brevican
BD: Bipolar disorder
CLD: C-type lectin domain
CRP: Complement regulatory protein
CSPGs: Chondroitin sulfate proteoglycans
ECM: Extracellular matrix molecules
EGF: Epidermal growth factor
GAG: Glycosaminoglycan
GWSA: Genome-wide significant association
NCAM: Neuronal cell adhesion molecule
NCAN: Neurocan
QSAR: Quantitative structure-activity relationship
PhAPs: Phenothiazines
SNP: Single-nucleotide polymorphism
VCAN: Versican.
